# Criteria-Based Decision Making for Introducing Open Kinetic Chain Exercise after-ACL Reconstruction: A Scoping Review

**DOI:** 10.1186/s40798-025-00843-8

**Published:** 2025-04-12

**Authors:** Florian Forelli, Jean Mazeas, Vasileios Korakakis, Haashim Ramtoola, Amaury Vandebrouck, Pascal Duffiet, Louis Ratte, Georgios Kakavas, Ismail Bouzekaroui Alaoui, Maurice Douryang, Andreas Bjerregaard, Jérôme Riera, Alexandre J. M. Rambaud

**Affiliations:** 1 Haute-Ecole Arc Santé, HES-SO University of Applied Sciences and Arts Western Switzerland, Delémont, Switzerland; 2 Orthopaedic Surgery Department, OrthoLab, Ramsay Healthcare, Clinic of Domont, Domont, France; 3 SFMK Lab, Pierrefite sur seine, France; 4 Department of Health Sciences, School of Life Sciences and Health Sciences, PhD in Physiotherapy Program, University of Nicosia, Nicosia, Cyprus; 5 Hellenic Orthopaedic Manipulative Therapy Education (HOMT Edu), Athens, Greece; 6 Fysiotek Spine & Sports Lab, Athens, Greece; 7 Department of Physical Education and Sport Sciences, ErgoMech-Lab, University of Thessaly, Volos, Greece; 8 Mohammed VI Center for Research and Innovation, Rabat, Morocco; 9 Mohammed VI Faculty of Nursing and Allied Health Professions, University of Sciences and Health, Casablanca, Morocco; 10 Department of Physiotherapy and Physical Medicine, University of Dschang, Dschang, Cameroon; 11 Physiotherapy Department, University of Southern, Odense, Denmark; 12 Inter- university Laboratory of Human Movement Biology, University Jean Monnet Saint-Etienne, University Savoie Mont-Blanc, Lyon 1, Saint-Etienne, 7424, F-42023 EA France; 13 College of Health Sciences, University of Bordeaux, IUSR, Bordeaux, 33000 France; 14 IFMK Saint Etienne, Saint Michel Campus, Saint Etienne, 42000 France

**Keywords:** Anterior Cruciate Ligament Reconstruction, Quadriceps Strengthening, Rehabilitation, Open Kinetic Chain

## Abstract

**Background:**

After an anterior cruciate ligament reconstruction (ACLR), mounting evidence suggests that open kinetic chain (OKC) strengthening is safe, reduces the risk of anterior knee pain, and significantly improves the quadriceps strength. However, clinicians are reluctant to use OKC knee strengthening exercises mainly due to the strong beliefs that they might increase graft laxity. The objective of this scoping review is to identify the key criteria employed in the scientific literature for the safe introduction of OKC quadriceps strengthening following ACLR.

**Methods:**

A scoping review of the literature was conducted on the online databases MEDLINE (PubMed), ScienceDirect, Embase and CINAHL Library online. Data regarding time-based criteria and/or clinical based criteria allowing OKC exercises introduction following ACLR were searched for. Only studies involving patients who performed quadriceps strengthening using any type of OKC exercises were included, regardless of the type, resistance location, load magnitude, type of muscle contraction, knee range of motion, or duration of the strengthening protocol.

**Results:**

Twenty-six studies met the inclusion criteria. Twenty-one employed time-based criteria for the introduction of OKC exercise. The median time from when OKC was permitted was 15 postoperative days (range 1–270 days), while the mean time was 31.6 ± 56.7 postoperative days. In 30.7% of the studies additional clinical examination components were used. These components included range of motion (0-100°), numeric pain scale score < 2 or 3, absence of joint effusion (assess by the stroke test), full knee active extension (assess by the straight leg raise), and walking without crutches for the decision-making regarding OKC exercise introduction.

**Conclusion:**

Less than one study in 3 reported clinical criteria for the introduction of OKC exercise. This highlights the absence of consensus among surgeons and physiotherapists, thereby hindering their ability to make informed decisions based on scientific evidence. Although the use of OKC exercise appears to be safe, precautions to maintain the integrity of the surgical repair need to be implemented. The establishment of valid criteria is crucial to support evidence-based decision-making.

## Key Points

Open kinetic chain (OKC) exercises are generally safe and can significantly improve quadriceps strength without increasing the risk of graft laxity after anterior cruciate ligament reconstruction (ACLR).

The majority of studies (80.7%) used time-based criteria for introducing OKC exercises, with a median time of 15 postoperative days.

Combining clinical and temporal criteria ensures safe and effective integration of OKC exercises in ACLR rehabilitation.

## Background

A key objective of sports injury rehabilitation is to ensure a safe and efficient return to sport (RTS) while minimizing the risk of re-injury and optimizing knee function after anterior cruciate ligament reconstruction (ACLR) [1, 2]. 

Early rehabilitation protocols often prioritize closed kinetic chain (CKC) exercises due to their safety profile, as they induce low graft stress by increasing tibiofemoral compression forces and hamstring activation while limiting anterior tibial translation [3]. However, CKC exercises are not optimal for isolating and strengthening the quadriceps, which is crucial for restoring knee function and reducing reinjury risk [4]. Despite strong evidence demonstrating that open kinetic chain (OKC) exercises do not compromise graft stability and significantly improve quadriceps strength, their use remains controversial in clinical practice. Concerns about graft laxity persist among clinicians, leading to inconsistent implementation of OKC exercises in ACLR rehabilitation. Recent studies indicate that a combination of CKC and OKC exercises can enhance quadriceps recovery, promote an earlier RTS, and improve functional outcomes without increasing anterior tibial laxity [4–7]. 

Biomechanical studies have shown that ACL strain during OKC leg extension (3.8%) is significantly lower than the strain experienced during normal walking (13%) [3, 8–10]. Furthermore, studies comparing CKC and OKC exercises early after ACLR consistently report superior isokinetic strength and endurance of the knee extensor muscles with OKC exercises at 3, 6, or 12 weeks postoperatively [6, 7, 11]. Current clinical practice guidelines support the safe use of OKC exercises even in early rehabilitation, emphasizing their role in improving quadriceps strength and expediting RTS without compromising knee stability [12]. 

Quadriceps strength is strongly associated with favorable outcomes, including improved gait, functional performance, quality of life, and reduced risk of re-injury and knee osteoarthritis [13]. Quadriceps strength recovery and symmetry between the operated and non-operated limb are essential criteria for progressing rehabilitation, return to running, and resuming sports activities [12, 14–16]. Since OKC exercises effectively target the quadriceps, their early implementation may improve recovery and the time to RTS [4, 11]. Despite these benefits, inconsistencies in research protocols—such as variations in graft types, sample sizes, intervention strategies, and outcome measures—limit the clinical applicability of existing evidence [4–7, 12]. Current guidelines lack standardized criteria for determining the appropriate timing for OKC exercise introduction, leading to variability in clinical practice.

The objective of this scoping review is to examine the temporal and/or clinical criteria employed in the scientific literature to determine the appropriate timing for the introduction of OKC exercises following ACLR.

## Methods

Scoping reviewers allow researchers to explore and map the extent of the current available evidence around a specific topic by using heterogeneous study designs [17, 18]. 

This review followed the methodology described by Levac et al. and the Joanna Briggs Institute methodology for scoping reviews, while the reporting has followed the Preferred Reporting Items for Systematic Reviews and Meta-Analyses Extension for Scoping Reviews (PRISMA-ScR) [19, 20]. 

The review is not registered a priori, and no patients or members of the public were involved.

### Eligibility Criteria

All types of clinical trials, preliminary studies, recommendations, rehabilitation protocols, and case studies written in English language were included [20]. We did not include literature reviews, systematic reviews and meta-analyses, as well as conference abstracts and editorials [20]. No restrictions were placed on the year of publication.

Only studies including patients who have undergone an ACLR using hamstring tendon or patellar tendon grafts (any other type of surgery was excluded), regardless of their sports activities and level were included. The age range of participants was limited to a minimum of 16 years and a maximum of 60 years. Patients who have undergone meniscectomy, had cartilage lesions or osteochondral defects, injury of the medial collateral ligament, posterior cruciate ligament injury, or ACL revisions were not included.

The present study included only studies involving patients who performed quadriceps strengthening using any type of OKC exercises (with and without resistance), irrespective of the type (manual resistance, leg extension machine, isokinetic, etc.), resistance location (proximal or distal tibia), load magnitude, type of muscle contraction, range of knee motion, or duration of the strengthening protocol. Studies with protocols that did not explicitly detail the OKC strengthening program used and did not specify graft type were not included.

### Information Sources, Literature Search, and Selection of Sources of Evidence

An exploratory search was performed (June 2024) in the MEDLINE database using the terms: ‘anterior cruciate ligament’ AND ‘reconstruction’ AND ‘open kinetic chain’. By this preliminary search we identified MeSH terms, text word terms, and relevant keywords, as well as search strategies from relevant systematic reviews, and in consultation with the author team we developed the final key word string to be used for the search (Table 1).

**Table 1:** Search terms

Construct	Keywords
Population	Anterior cruciate ligament reconstruction, Anterior cruciate ligament reconstruction graft, Anterior cruciate ligament reconstruction surgery
Concept	Open kinetic chain, open chain, isokinetic, leg extension, open kinematic chain
Context	Muscular strengthening, resistance training, strength training, strengthening program

Relevant studies were identified by the lead author by systematically searching four online databases from inception to June 2024 (MEDLINE via PubMed, Embase, ScienceDirect, and CINAHL). The reference lists of included studies, along with key systematic and narrative reviews, were screened. Additionally, citation tracking was performed using Google Scholar to identify any relevant studies that might have been missed during the database search.

**Fig. 1:**
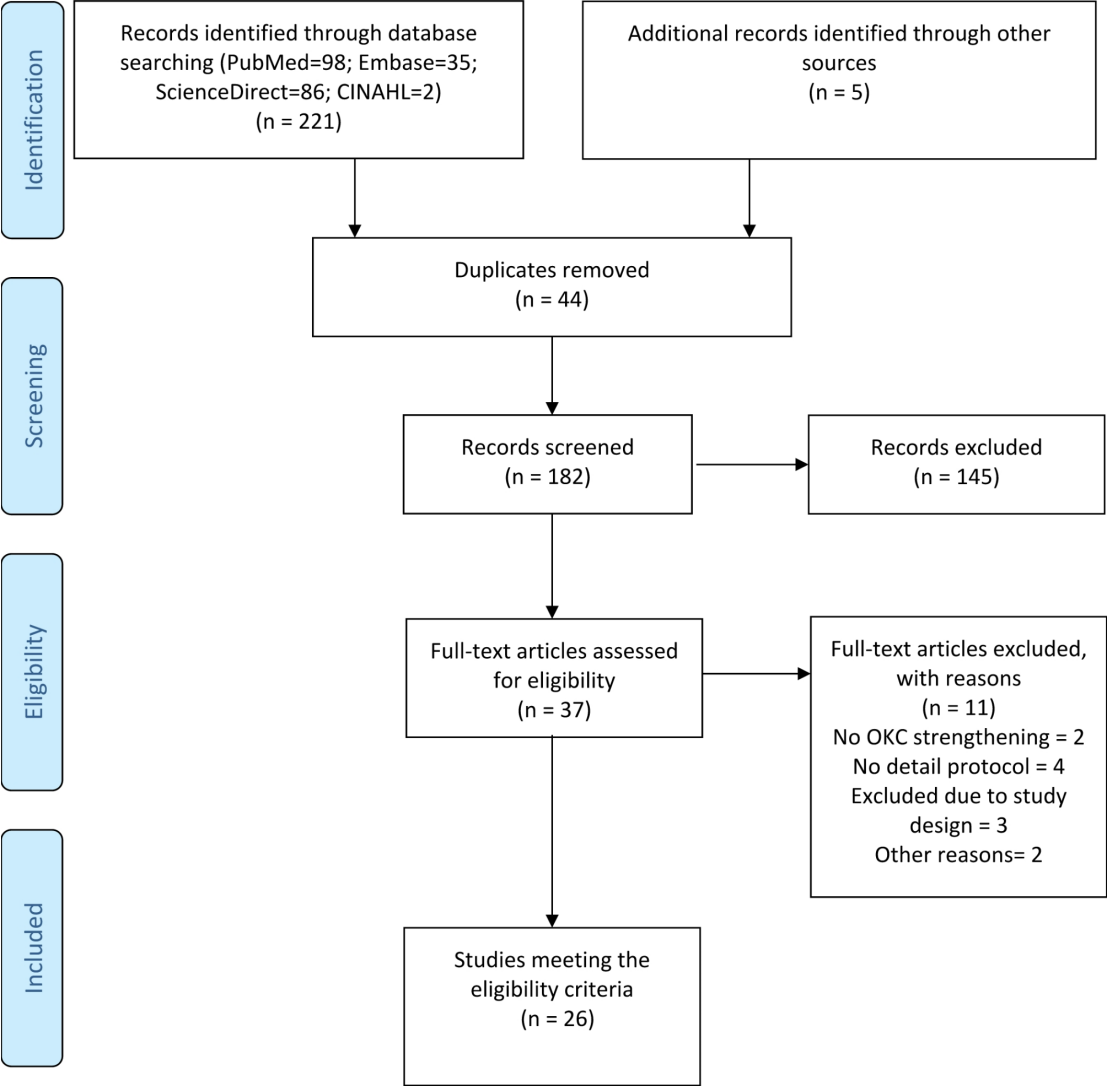
Preferred Reporting Items for Systematic Reviews and Meta-Analyses Extension for Scoping Reviews flow chart

All articles were downloaded and transferred to Zotero v6.0.13 management platform. Cross-referenced and any duplicates were deleted before the selection criteria were applied. Two reviewers (HR and FF) independently screened all articles for eligibility by title and abstract (Fig. 1). The same independent reviewers (HR and FF) conducted the full-text screening to finalize study selection. Any discrepancies were first discussed in a consensus meeting between the reviewers. If consensus was not reached, the senior author (AJM, VK, JR) was consulted to resolve the disagreement.

### Data Extraction

A standardized data extraction Excel sheet was developed and subjected to a pilot test on a sample comprising 10% of the eligible studies. Extracted data included: article identifiers (authors, title, year of publication, country, and study design), graft type, time-point of OKC exercise initiation, criteria used to initiate OKC exercises, type of OKC exercise (ROM, contraction mode, load and progression).

Authors of studies reporting or suggesting OKC exercises for quadriceps strengthening without any detail of criteria for initiation were contacted via email (twice). If no response was received, these studies were excluded from the scoping review (Fig. 1).

### Critical Appraisal of Individual Sources of Evidence

Given that we conducted a descriptive analysis of variables (time-based criteria and clinical criteria) representing the rehabilitative strategy used in the included studies, and not the results of an intervention administered to a population, we argue that our descriptive summary is less likely to be negatively influenced by the quality or the risk of bias of the studies.

### Data Management and Synthesis

Data were presented either as counts and percentages (%), median and interquartile range (IQR), or mean and standard deviation (SD); due to the lack of consensus on the appropriate timing for initiating OKC exercises in ACLR rehabilitation, steps were taken to ensure that the results were not disproportionately influenced by outliers. If a study compared two rehabilitation protocols with different time-based or other criteria, all criteria were included in the summary.

## Results

An overview of the study identification and selection process is depicted in Fig. 1. Of 226 potential records, 182 unique records underwent title/abstract screening, 37 were reviewed in full, and 26 studies were included.

Twenty-one eligible studies (*n* = 21; 80.7%) used or included time-based criteria to introduce OKC quadriceps strengthening (IQR = 15 days– range 1 to 270 days; mean ± SD 31.6 ± 56.7 days) (Table 2). One-third of these studies (*n* = 9; 34.6%) introduced OKC strengthening between the 7th and the 30th post-operative day, while six studies (*n* = 6; 23.1%) suggested or introduced OKC quadriceps strengthening in the first postoperative week (range 1–7 days). Twelve studies (*n* = 12; 46.1%) included bone patellar-tendon bone graft and 14 studies (*n* = 14; 53.8%) included hamstring graft. The timing of OKC exercise introduction for bone patellar-tendon bone graft was 42.8 ± 74.1 days and 35.5 ± 38.8 days for hamstring graft.

**Table 2:** An overview OKC exercise introduction for quadriceps strengthening following an ACL reconstruction

Study	Country	Graft type	Timing and/or criteria of OKC	Exercise(s) (frequency)	Details and progressions of OKC exercise	Adverse events	Results
Beynnon et al., 2011 (RCT) [59]	United States	BPTB	15th post-op day	SLR (2–3 daily) Leg extension (2–3 daily)	ROM 45–90° (10 reps x 3 sets) without external load at 2 weeks ROM 0–90° (10 reps x 3 sets) with external load (5 lbs) at 5 weeks ROM 0–30° - Short Arc Quad (10 reps x 3 sets) without external load at 5 weeks ROM 0–90° (10 reps x 3 sets) with external load (unlimited) at 6 weeks	NR	Both accelerated and non-accelerated rehabilitation programs led to a similar increase in the envelope of knee laxity. The majority of the knee laxity increase occurred during the healing phase when exercises were advanced, and activity levels increased. Patients in both programs demonstrated equivalent outcomes Despite these improvements, participants in both treatment groups did not achieve pre-injury levels on the KOOS assessment of quality of life.
Buckthorpe et al., 2020 (Clinical Commentary) [60]	United Kingdom & Italy	BPTB & HS	o No crutches o ROM 0-120° o No effusion o Full quadriceps activation	NA	First half of mid stage: Low to moderate load and restricted ROM 50–90° Second half of mid stage: Load at 8–12 RM and full ROM	NR	NA
Bynum et al., 1995 (Prospective RCT) [61]	United States	HS	21st post-op day	Quadriceps Isometric contraction Quadriceps Isotonic leg extension	Quadriceps Isometric contraction at 3 weeks Quadriceps Isotonic leg extension at 6 weeks with low load Unrestricted quadriceps eccentric and concentric contraction at 12 weeks	NR	OKC exercises, when incorporated into an accelerated rehabilitation protocol, are a safe and effective method for early-stage knee rehabilitation after ACLR.
Cristiani et al., 2020 (RCT) [62]	Sweden	BPTB & HS	45th post-op day	Isokinetic leg extension (concentric and eccentric mode)	Restricted ROM 0–90° (NS sets-reps) at 4 weeks Full ROM at 3 months	NR	Muscle strength and SLH test performance improved progressively after ACLR. Inferior quadriceps strength was observed in the BTPB group compared to the HS group at 4, 6, 8, and 12 months postoperatively. Poorer SLH test performance was found in the BTPB group compared to the HS group at 4 months postoperatively. Persistent deficits in hamstring strength were observed at all postoperative follow-ups in the HS group.
Ebert et al., 2022 (RCT) [63]	Australia	HS	21st post-op day	Isometric leg extension Isotonic leg extension	Isometric knee extension (multi-angle: at 90°, 60° and 45°) Isotonic knee extension at restricted ROM 45–90° without external load at 3 weeks Isotonic leg extension a full ROM, without external load at 4 weeks	NR	Accelerated rehabilitation pathway could enhance strength and functional symmetry after ACLR which have been linked with re-injury upon RTS, without affecting laxity. High levels of patient satisfaction and RTS rates were observed in both group
Elabd et al., 2023 (RCT) [64]	Egypt	HS	1st post-op day	SLR Leg extension	10 rep. x 3 sets X 4–5 times/day 90^◦^–40^◦^ Week 4–8 15 rep. x 3 sets AROM No weight added 90^◦^–0^◦^ Week 8–13 × 3 sets AROM No weight added Full Starting at week 13 15 rep. x 3 sets Low resistance (manually or by theraband) OKC quadriceps exercises(Leg extension) 90 − 45^◦^ Week 4: 15 rep. x 3 set No weight added 90^◦^ − 30^◦^ Week 5 90^◦^ − 20^◦^ Week 6 90^◦^ − 10^◦^ Week 7 90^◦^ − 0^◦^ Week 8 OKC exercises to full ROM (Leg extension)at the start of phase 2. No weight was added Week 12 20 rep. x 3 set (30 RM)	NR	Greater reduction of knee pain and effusion and better normalization of knee functions than conventional program
Forelli et al., 2023 (Cohort study) [6]	France	HS	VAS < 2 SLR without lag ROM 0-100 Stroke Test < 1+ GNRB at 134 *N* < 1.5 mm	Leg extension Isokinetic Leg extension with external load	10 sets of 8 repetitions at 60 ^◦^/s 8 sets of 8 repetitions (Leg extension 3 times per week 1 s of isometric contraction, and 3 s of eccentric contraction 60% RM	NR	Quadriceps strength Improvement at 3 and 6 months without graft laxity increasing at 3 and 6 months
Forelli et al., 2024 (Cohort study) [7]	France	HS	VAS < 2 SLR without lag ROM 0-100 Stroke Test < 1+ GNRB at 134 *N* < 1.5 mm	Leg extension Isokinetic Leg extension at 2 weeks without external load Leg extension with external load when the patient meet criteria	10 sets of 8 repetitions at 60 ^◦^/s 8 sets of 8 repetitions (Leg extension 3 times per week 1 s of isometric contraction, and 3 s of eccentric contraction 60% RM	NR	Quadriceps strength Improvement at 3 months without graft laxity increasing at 3 and 6 months
Fukuda et al., 2013 (RCT) [65]	Brazil	HS	30th post-op day	Isometric leg extension + NMES Isotonic leg extension + NMES	Isometric leg extension + NMES: ROM 60°, 10 reps x 10s Isotonic leg extension + NMES: ROM 45–90°, 3 × 10 reps from 4 to 12 weeks ROM 0–90°, 3 × 10 reps from 12 weeks	NR	Starting OKC exercises for quadriceps strengthening at 4 weeks postoperatively (with a restricted range of motion of 90°–45°) showed no difference in anterior knee laxity compared to starting at 12 weeks postoperatively. Both early (4 weeks) and late (12 weeks) OKC exercise groups achieved similar results in terms of pain reduction and Functional improvement. The early OKC exercise group demonstrated a faster recovery of quadriceps strength compared to the late OKC group
Heckmann et al. 2017 (Clinical Commentary) [66]	United States	BPTB	1st post-op day	SLR Isotonic leg extension	SLR: 3 sets x 10 reps without external load until 8 weeks 3 sets x 10 reps with external load (10% BW) at 5 weeks Isotonic leg extension: restricted ROM 30–90°, 3 sets x 10 reps without external load Restricted ROM 30–90°, 3 sets x 10 reps with external load at 5–6 weeks Restricted ROM 30–90°, 1–2 sets x 8–12 reps with external load at 27 weeks	NR	NA
Heijne et al., 2006 (Prospective RCT) [67]	Sweden	BPTB & HS	30th post-op day	Isotonic leg extension	Restricted ROM 40–90° at 4 weeks without external resistance ROM 0–90° at 5 weeks without external resistance ROM 0–90° at 6 weeks with unlimited external resistance according to symptoms and tolerance of each patient	NR	Accelerated rehabilitation pathway could enhance strength and functional symmetry after ACLR which have been linked with re-injury upon RTS, without affecting laxity.
Hooper et al., 2001 (RCT) [68]	United Kingdom	HS	Between 12th and 19th post-op day ROM 0–90° No crutches	Isotonic leg extension (1.5 s concentric, 3 s eccentric)	ROM 0–90°, 3 × 20 reps (RM)	NR	Gains in strength after resistance training of the knee extensors following ACLR surgery were related of the knee joint angle. Training the affected leg after surgery resulted in no substantial increases in knee extensor strength at both test velocities, 60°/s and 210°/s, with the greatest gains found in the middle of the ROM.
Hooper et al., 2001 (RCT) [69]	United Kingdom	HS	15th post-op day	Isotonic leg extension (1.5 s concentric, 3 s eccentric)	ROM 0–90°, 3 × 20 reps (RM)	NR	No clinically significant differences in the functional improvement resulting from the choice of open or closed kinetic chain exercises in the early period after this surgery.
Lutz et al., 1990 (Clinical commentary) [70]	United States	BPTB & HS	270th post-op day	Isotonic leg extension	Restricted ROM 30–90°	NR	NA
Mikkelsen et al., 2000 (Prospective RCT) [71]	Sweden	BPTB	45th post-op day	Isokinetic leg extension (concentric and eccentric mode)	Restricted ROM 40–90° (120 + 30°/s − 50 reps) at 6 and 7 weeks Restricted ROM 40–90° (120 + 30°/s − 80 reps) at 7 weeks Restricted ROM 30–90° (120 + 30°/s; 90 + 90°/s − 60 + 60 reps, respectively) at 8 weeks Restricted ROM 30–90° (120 + 30°/s; 90 + 90°/s − 90 + 80 reps, respectively) at 9 weeks ROM 20–90° (120 + 30°/s; 90 + 90°/s; 30 + 120°/s − 3 × 70 reps) at 10 weeks Restricted ROM 20–90° (120 + 30°/s; 90 + 90°/s; 30 + 120°/s − 2 × 80 + 90 reps, respectively) at 11 weeks Restricted ROM 20–90° (120 + 30°/s; 90 + 90°/s; 30 + 120°/s; 240 + 240°/s − 80 + 70 + 80 + 70 reps, respectively) at 12 weeks	NR	Combination of CKC and OKC quadriceps exercises is more effective than using CKC exercises alone after ACL reconstruction. Combination of CKC and OKC exercises leads to significantly better quadriceps muscle torque. Combination of CKC and OKC exercises enables a significantly earlier return to original sports at the same pre-injury level. Combination of CKC and OKC exercises does not compromise knee joint stability.
Morrissey et al., 2000 (RCT) [48]	United Kingdom	BPTB	15th post-op day	Isotonic leg extension (1.5 s concentric, 3 s eccentric)	ROM 0–90°, 3 sets x 20 reps (RM)	NR	OKC training offers an advantage over closed kinetic chain knee extensor training after ACLR, closed kinetic chain training is the treatment of choice.
Morrissey et al., 2000 (RCT) [72]	United Kingdom	BPTB	15th post-op day ROM 0–90° No crutches	Isotonic leg extension (2 s concentric, 2 s eccentric)	ROM 0–90°, 3 sets x 20 reps (RM)	NR	OKC resistance training velocity specificity appears to occur only in the later phase of rehabilitation using isokinetic exercise.
Morrissey et al., 2003 (RCT) [47]	United Kingdom	BPTB	15th post-op day ROM 0–90° No crutches	Isotonic leg extension (2 s concentric, 2 s eccentric)	ROM 0–90°, 3 sets x 20 reps (RM)	NR	Knee laxity and function are not affected differently by CKC and OKC training of the knee and hip extensors
Perry et al., 2005 (RCT) [73]	United Kingdom	BPTB	60th post-op day	Isotonic leg extension (1.5 s concentric, 3 s eccentric)	ROM 0–90°, 3 sets x 20 reps (RM) from 4 to 6 weeks 3 sets x 6 reps (RM) at 6 weeks Increase load if pain < 5	NR	CKC and OKC training regimens, with the loads used in this study, showed no differences in their effects on knee laxity and knee function during the 8- to 14-week postoperative period after ACLR. OKC training appeared to be safe with the training loads used in this study OKC training did not demonstrate any particular advantages over CKC training for patients following ACLR.
Shaw et al. ; 2005 (RCT) [74]	Australia	BPTP & HS	1st post-op day	Isometric quadriceps contraction SLR	10 repetitions, 3 times daily till 2 weeks	NR	Subjects had faster recovery in range of motion and some had higher Cincinnati Knee Rating System scores No quadriceps strength and lower limb function improvement
Uçar et al., 2014 (RCT) [75]	Turkey	HS	3rd post-op day	SLR Leg extension	SLR, ROM 0–50°, 3 sets x 20 reps Leg extension: ROM 0–90°, 3 sets x 20 reps (when knee flexion was 110°)	NR	CKC exercises are more effective than OKC exercises, at providing mobilization and enabling a quicker return to daily and sporting activities.
Vidmar et al., 2019. (RCT) [76]	Brazil	HS	30th post-op day	Isokinetic eccentric training	Isokinetic eccentric training program was applied for 6 weeks, twice a week, with a minimal interval of 72 h between sessions. In each training session, participants performed three (1st mesocycle: weeks 1—3) or four sets (2nd mesocycle: weeks 4—6) of 10 maximal intensity knee extensor eccentric contractions	NR	OKC eccentric training is a safe and effective strategy for rehabilitating the quadriceps muscle after ACL reconstruction Isokinetic eccentric training produced greater improvements in quadriceps muscle mass and strength
Welling et al., 2019 (non-randomised controlled study) [49]	Netherlands	BPTB & HS	VAS < 3/10 No knee joint effusion Full quadriceps activation	Isotonic leg extension	Leg extension: restricted ROM 45–90° (2 sets x 15–25 reps - < 50%RM) Increase ROM progressively to 2–4 sets x 8–10 reps at 60 − 80% RM) 4 sets of 4-12-10-8 reps	NR	Progressive strength training in rehabilitation after ACLR may mitigate commonly reported strength deficits
Wilk et al., 2017 (Clinical commentary) [77]	United States	BPTB & HS	2nd post-op day	Isometric leg extension Isotonic leg extension	Multiangle isometric leg extension between 60–90° Leg extension: restricted ROM 40–90°	NR	NA
Yabrudi et al. 2013 (Narrative review) [50]	United States	BPTB & HS	Walking without crutches Knee extension like non-operated limb ROM 0-120° Full knee active extension No knee joint effusion Full quadriceps activation	Isotonic leg extension	Restricted ROM 60–90° for the first 3 months Full ROM after 3 months	NR	NA
Yasuda et al., 1995 (Prospective RCT) [78]	Japan	HS	7th post-op day	Isometric knee contraction Isotonic leg extension Isokinetic knee extension	Isometric knee contraction: 70° at 1 week Isotonic leg extension: ROM 0-120° at 2 weeks ROM 0–140° at 4 weeks Full ROM at 8 weeks Isokinetic knee extension: ROM 30–90° at 8 weeks Full ROM at 6 months	NR	No significant difference in the isometric quadriceps strength between the two groups at each postoperative period Isometric hamstring muscles strength in the knees with both the reconstruction and the harvest surgeries was significantly lower than that in the knees with only the reconstruction surgery only at 1 month postoperatively No significant difference in the postoperative laxity between the two groups.
Abbreviations: NA, non-applicable; NS, not stated; NR: not reported, RCT, randomized controlled trial; ROM, range of motion; OKC, Open Kinetic Chain; RM, Repetition maximum; SLH; Single Leg Hop; SLR, Straight Leg Raise; D; BPTB, Bone Patellar Tendon Bone graft; HS, Hamstring graft; reps, repetitions; VAS, Visual Analogic Scale; KOOS, Knee Injury and Osteoarthritis Outcome Score; IKDC, International knee documentation committee; ACL RSI, Anterior Cruciate Ligament– Return to Sport and Injury; SLVJ, Single Leg Vertical Jump							

Only three studies (*n* = 3; 11.5%) used both time-based and clinical criteria to introduce OKC quadriceps strengthening. Two of these (*n* = 2) included ACL patients with a bone patellar-tendon bone graft (Table 2). All three studies (*n* = 3) introduced OKC strengthening around the second post operative week (12–19 days). Regarding the clinical criteria, every study specified the knee range of motion as a required criterion along with walking without crutches. Two studies specified that the ACLR patient should have achieved full knee extension (0°) and flexion at 90°, while one study (*n* = 1) only mentioned knee flexion range of motion (90°) as a requirement for the introduction of OKC strengthening.

Five studies (*n* = 5; 19.2%) only used clinical criteria to allow the introduction of OKC quadriceps strengthening after an ACLR (Table 2). They all (*n* = 26) included ACL patients with mixed bone patellar-tendon bone and hamstring graft. The criteria used included full quadriceps activation (full knee extension with superior patellar glide), unassisted walking, minimal or absence of knee joint effusion, 120° of knee flexion active range of motion (ROM) and absence of knee laxity. Three studies (*n* = 3) used the presence of pain as a criterion and set a threshold of < 2 or 3/10 on a numeric pain rating scale for OKC exercise introduction.

## Discussion

This scoping review included 26 studies and revealed that time after surgery was the most frequently reported criterion for allowing the introduction of OKC quadriceps strengthening exercises after ACLR. From 21 studies (*n* = 21) it was found that the median time for the introduction of OKC exercise in rehabilitation was the 15th post-operative day (mean time was 31.6 ± 56.7 days), with a wide range of initiation dates, from as early as the first post-operative day to as late as nine months after the ACLR. Five out (*n* = 5) of the 26 included studies reported or suggested clinical criteria for the introduction of OKC exercise, with knee flexion ROM > 100°, minimal or absence of joint effusion, normalized unsupported gait, and normal quadriceps activation being the most frequently reported.

### OKC Exercise Introduction, Clinical Practice, and Guidelines

Previous and current clinical practice guidelines on rehabilitation after ACLR suggest delaying OKC quadriceps strengthening exercise until 4 weeks post-operatively in a restricted range of knee motion (90°−45° of knee flexion) [12, 21]. These guidelines and the findings of our review are aligned with current physiotherapy practices in Europe, which indicate that among practitioners who use OKC exercises in ACLR rehabilitation, the majority introduce these exercises before six weeks post-operatively, with only around 20.0% initiating these types of exercises after 12 weeks [22, 23]. A survey of Greek physiotherapists showed that most of them (64.3%) seemed to introduce OKC exercises based on a combination of clinical criteria (i.e., graft type, knee swelling and pain) and time since surgery [23]. On the contrary, 61.7% of Flemish physiotherapists do not use OKC exercises during ACLR rehabilitation [22]. This approach may stem from the traditional belief that OKC knee extension exercises are dangerous for the ACL graft, or may reflect a more conservative approach [4]. Nevertheless, clinical practice guidelines and current clinical practices do not align with contemporary evidence of no difference in anterior tibial laxity, quadriceps strength, patient-reported function, or physical function with early or late introduction of OKC exercises in the ACLR population, when compared to closed kinetic chain exercises [3, 6, 7]. Finally, despite guidelines being more conservative regarding the introduction of OKC exercises in ACLR patients with a hamstring graft, this was not reflected in the present review with most studies introducing OKC exercises early in the rehabilitation process, with a restricted knee range of motion, but allowing external resistance (Table 2).

### Introducing OKC Quadriceps Exercises Solely Based on Time After ACLR is not Relevant in Clinical Practice

Contemporary evidence and clinical practice guidelines on rehabilitation after ACLR conclude in favor of discarding temporal data dictating rehabilitation and assert that rehabilitation consists of several phases based on criteria and specific goals and objectives rather than time [12, 21, 24, 25]. Nevertheless, these recommendations still use a time-based criterion (fourth week) for the introduction of OKC quadriceps exercises, as did 80.7% of the included studies in the present scoping review. The understanding of the stages– especially their duration– of the ACL graft healing process is still limited [26]. Based on data from imaging and biopsy studies, it has been argued that the graft healing phase lasts for 3 months including a period of inflammation and cell necrosis (3 to 6 weeks), the consolidation of bone-to-bone interface takes 3 months when using the bone-patellar tendon-bone as a graft, and the healing of bone tunnels is even longer (more than a year) in the case of a reconstruction using a hamstring graft [27, 28]. Low stiffness of the ACL graft could therefore be an indication that the maturation of the ACL graft is not sufficient and that the tensile strength has not recovered. In a prospective study by Lindanger et al., the authors proposed a categorization of knee laxity measured with a KT1000 arthrometer. A side-to-side difference of < 3 mm was defined as ‘tight’, 3 mm to 5 mm was defined as ‘slight loss’, and > 5 mm was defined as ‘loose graft’. Furthermore, the authors suggested that a cut-off point of < 3 mm of anterior translation could be used as a RTS criterion [29]. These data plausibly, along with some of the earliest reports warning against the use of open-chain exercises, feed the “fear” in the clinical community and facilitate the building of strong beliefs that OKC exercises are unsafe and result in loosening of the ACL graft stiffness, all against overwhelmingly contradictory evidence [4–7, 30]. However, the tensile strength of the ACL graft during the maturation phase after ACLR remains unknown.

### The Role of Mechanical Loading in ACLR Rehabilitation

From a different perspective, the adaptation of biological tissues, including tendons, to mechanical loading is a fundamental principle of rehabilitation. Tendons, such as those used in ACL grafts, undergo hypertrophic and structural adaptations in response to controlled loading, highlighting the importance of appropriate mechanical stress during recovery [31–33]. Early and progressive loading has been shown to promote optimal tissue remodeling and functional recovery, as it stimulates cellular activity and collagen synthesis, thereby enhancing the mechanical properties of the graft [34, 35]. 

In the context of ACLR, the loading introduced through OKC exercises is not excessive and falls within a range that supports graft adaptation [36–39]. These exercises provide a targeted approach to load the graft while isolating the quadriceps, aiding in muscle recovery and preventing long-term deficits in quadriceps function and morphology [40–43]. Emphasizing the benefits of early, controlled loading could strengthen the argument for the safe inclusion of OKC exercises in rehabilitation protocols, demonstrating their dual role in promoting graft health and restoring muscle strength [44]. Given that the graft as a living tissue reacts to the imposed mechanical stress and that OKC exercises impose similar stress to gait, we could also assume that early mechanical load (tension) at the graft should promote the healing response and improve its mechanical properties as seen in the ACL pre-to-post season comparison of healthy competitive athletes [45, 46]. We also must acknowledge the muscle contribution to resist anterior translation of the tibia relative to the femur, and it has been suggested that interventions aiming to mitigate risk of ACL injury consider targeting the function of these specific muscles [36]. Furthermore, tensile stress would mostly be relative to age, with older individuals having lower tensile strength [36]. 

### A Criteria-Based Approach for OKC Quadriceps Exercise Introduction

We respectfully suggest that while time-based criteria for introducing OKC quadriceps strengthening exercises have traditionally been used, their relevance in clinical practice may be limited, and they may not fully achieve their intended safety objectives. Consistent with an individualized clinical reasoning approach, we propose that a criteria-based framework, tailored to the specific needs and progress of each ACL-reconstructed patient, should guide rehabilitation, including the introduction of OKC quadriceps exercises [12]. The studies in this scoping review (Table 2) indicated as criteria for introducing OKC exercises normalized unsupported gait, no or minimal knee joint effusion, knee flexion range of motion ≥ 90°, normal quadriceps activation or no lag during active straight leg raise [47–50]. From a clinical perspective, all these criteria could be supported by direct or indirect evidence of usefulness and applicability. Abnormal knee flexion would not allow for free OKC knee extension exercises, while knee flexion deficit at early follow-up has been implicated as a significant factor related to the presence of osteoarthritis on radiographs [51]. Normalized gait would allow the ACL graft to be introduced to the normal required stress during activities of daily living before the initiation of OKC exercises [52]. Lack of full quadriceps activation due to arthrogenic muscle inhibition may hinder OKC quadriceps strengthening and has been associated with substantial quadriceps weakness even early after injury [53–55]. However, only one of the included studies in this review followed a pain monitoring approach in the application of OKC exercises, despite current clinical practice guidelines indicating the clinician should monitor for anterior knee pain and adjust the OKC knee load and the progression of strengthening accordingly [12, 49]. It has not yet been elucidated how much pain is acceptable during exercising, despite the reasonable argument that pain experience related to exercise is likely important and may be an essential part of recovery, given that pain during exercise has not been found to be a barrier to successful outcomes and could contribute to superior clinical outcomes [56, 57]. 

### Methodological Considerations

To our knowledge, this scoping review is the first to focus on the introduction of OKC quadriceps strengthening exercises in patients after ACLR. However, several limitations must be acknowledged, as they significantly impact the generalizability of our findings.

First, our inclusion criteria did not account for ACLR using a quadriceps tendon graft, despite recent evidence and recommendations supporting its use. Given that graft type may influence rehabilitation timelines and protocols, the absence of studies focusing on quadriceps grafts limits the applicability of our results to this patient population. Future studies should investigate whether rehabilitation timelines and clinical criteria differ between hamstring, bone-patellar tendon-bone, and quadriceps tendon grafts.

Second, some studies were excluded from our review because their rehabilitation protocols were insufficiently detailed, preventing their inclusion in our analysis. Although we attempted to contact the authors for clarification regarding their protocols and the timing of OKC exercise introduction, we were unable to obtain this information. This may have resulted in a selection bias, as only studies with explicitly reported OKC exercise implementation were included. Consequently, our findings may not fully represent all rehabilitation approaches used in clinical practice.

Furthermore, we assume that publications focusing on OKC exercises may tend to introduce them earlier than what is commonly practiced across all ACLR rehabilitation protocols. If this assumption holds, it suggests that our findings may overestimate the prevalence of early OKC exercise introduction in standard clinical settings. This potential bias highlights the need for broader investigations into how rehabilitation protocols vary in different clinical environments, particularly in regions where conservative approaches remain predominant.

Finally, our search strategy may have been limited by the possible omission of key search terms, as no librarian was involved in the development of the search string. While we aimed to construct a comprehensive and systematic search strategy, the lack of specialized input in search term selection may have affected the completeness of our literature retrieval. This limitation underscores the importance of refining search methodologies in future reviews to ensure a more exhaustive capture of relevant studies.

Despite these limitations, our review provides valuable insights into the variability of OKC exercise introduction in ACLR rehabilitation and highlights the need for standardized clinical criteria. Future research should address these limitations by incorporating a wider range of graft types, refining search strategies, and investigating real-world rehabilitation practices to improve the generalizability of findings to broader clinical settings.

### Clinical Implications

The findings of this scoping review suggest that clinical criteria can be valuable in guiding the introduction of OKC quadriceps strengthening exercises in ACL rehabilitation. Clinical markers such as a pain score < 2–3, knee ROM (0–100°), absence of knee joint effusion, walking without crutches, and full knee extension provide practical benchmarks for tailoring rehabilitation protocols to individual patient needs. These criteria align with previous research emphasizing a shift away from rigid, time-based rehabilitation approaches in favor of a more functional, criteria-based framework [5, 58].

Introducing OKC exercises with a restricted range of motion (e.g., 90°–45°) at earlier postoperative stages appears to be safe and may contribute to improved quadriceps strength without negatively impacting graft integrity. This is particularly relevant given the role of mechanical loading in ACL graft adaptation. Evidence suggests that controlled early loading can promote tissue remodeling, stimulate collagen synthesis, and enhance the mechanical properties of the graft without compromising stability [36–39]. The similarity between the mechanical stress imposed by OKC exercises and that experienced during normal gait further supports their safe integration into rehabilitation [44].

Moreover, the absence of significant differences in anterior tibial laxity, quadriceps strength, patient-reported function, or physical function between early and late OKC exercise introduction reinforces the idea that delaying OKC exercises may not be necessary when clinical criteria indicate readiness [3, 6, 7]. In fact, the selective activation of the quadriceps in OKC exercises may play a crucial role in addressing arthrogenic muscle inhibition and preventing long-term quadriceps deficits, which are associated with delayed recovery and an increased risk of secondary ACL injury [40–43].

Clinicians are encouraged to consider both time-based and clinical markers to individualize rehabilitation, while closely monitoring patient progress and adjusting protocols as necessary. The use of clinical criteria may allow for a more personalized and adaptable rehabilitation process, ensuring that patients progress based on functional recovery rather than arbitrary timeframes. Additionally, considering the potential benefits of progressive mechanical loading on graft maturation, OKC exercises introduced within safe parameters may contribute to both muscle strength gains and overall graft health [31–35].

Although these findings provide useful insights, further research is needed to validate the proposed clinical criteria and establish more standardized guidelines. The variability in current rehabilitation practices and the persistence of conservative approaches despite strong supporting evidence highlight the need for high-quality studies to confirm the long-term safety and efficacy of early OKC exercise introduction. A cautious and evidence-informed approach is recommended to ensure optimal patient outcomes while maintaining graft safety. Expanding the understanding of how different rehabilitation strategies influence graft adaptation and patient function will be essential for refining rehabilitation protocols and optimizing return-to-sport outcomes [5, 58].

## Conclusion

This scoping review analyzed 26 studies on the introduction of OKC exercises after ACLR. While time-based criteria were the most commonly reported, fewer than half of the studies incorporated clinical markers such as knee range of motion (0–100°), pain score < 2–3, absence of joint effusion, full knee extension, and independent walking. The lack of standardized criteria limits evidence-based clinical decision-making.

Clinicians should consider both clinical and time-based criteria to personalize rehabilitation, ensuring safe and effective quadriceps strengthening while monitoring patient progress. Progressive loading strategies may further support graft adaptation and optimize recovery.

Future research should aim to establish standardized guidelines by investigating the impact of early vs. delayed OKC exercise introduction, different graft types, and progressive OKC loading on quadriceps function and knee stability. A structured, individualized approach can help ensure safer rehabilitation and more consistent clinical outcomes.

## Data Availability

The data presented in this study are available on request from the corresponding author. The data are not publicly available due to privacy restrictions.

## Abbreviations

ACL: Anterior Cruciate Ligament
ACLR: Anterior Cruciate Ligament Reconstruction
CKC: Closed Kinetic Chain
OKC: Open Kinetic Chain
ROM: Range of Motion
RTS: Return To Sport
